# Cholesterol crystals enhance TLR2- and TLR4-mediated pro-inflammatory cytokine responses of monocytes to the proatherogenic oral bacterium *Porphyromonas gingivalis*

**DOI:** 10.1371/journal.pone.0172773

**Published:** 2017-02-24

**Authors:** Tania Køllgaard, Christian Enevold, Klaus Bendtzen, Peter R. Hansen, Michael Givskov, Palle Holmstrup, Claus H. Nielsen

**Affiliations:** 1 Institute for Inflammation Research, Center for Rheumatology and Spine Disease, Rigshospitalet, Copenhagen University Hospital, Copenhagen, Denmark; 2 Department of Cardiology, Copenhagen University Hospital Gentofte, Copenhagen, Denmark; 3 Costerton Biofilm Center, Department of International Health, Immunology and Microbiology University of Copenhagen, Copenhagen, Denmark; 4 Section for Periodontology, Microbiology and Community Dentistry, Department of Odontology, Faculty of Health and Medical Sciences, University of Copenhagen, Copenhagen, Denmark; New York Medical College, UNITED STATES

## Abstract

Cholesterol deposits and pro-inflammatory cytokines play an essential role in the pathogenesis of atherosclerosis, a predominant cause of cardiovascular disease (CVD). Epidemiological evidence has linked periodontal disease (PD) with atherosclerotic CVD. Accordingly, viable periodontal pathogens, including *Porphyromonas gingivalis*, have been found in atherosclerotic plaques in humans and mice. We aimed to determine whether cholesterol crystals (CHCs) and oral bacteria synergize in the stimulation of human monocytes. Incubation of human monocytes with CHCs induced secretion of interleukin (IL)-1β, tumor necrosis factor (TNF)-α, IL-6, and IL-8. Moreover, CHCs markedly enhanced secretion of IL-1β by monocytes stimulated with the toll-like receptor (TLR) 4 agonist *Escherichia coli* lipopolysaccharide (LPS), and the TLR2 agonist *Staphylococcus aureus* lipoteichoic acid. Notably, CHCs also enhanced IL-1β secretion induced by *P*. *gingivalis* LPS and IL-1β secretion induced by whole *P*. *gingivalis* bacteria. This enhancement was abrogated by the NLRP3 inflammasome inhibitors Z-YVAD-FMK and glibenclamide. CHCs had no effect on cytokine production induced by *P*. *gingivalis* gingipains. Taken together, our findings support that CHCs, via stimulation of NLRP3 inflammasomes, act in synergy with the periodontal pathogen *P*. *gingivalis* to promote monocyte secretion of pro-atherogenic cytokines.

## Introduction

Growing evidence suggests that periodontal disease (PD) is a risk factor for atherosclerotic cardiovascular disease (CVD) [1,2]. Atherosclerosis is generally accepted to be a chronic inflammatory disease in which the pro-inflammatory cytokines interleukin (IL)-1β and tumor necrosis factor (TNF)-α play an important role [3,4]. Accumulation of cholesterol crystals (CHCs) is thought to play an important role in atherosclerotic plaque destabilization and consequent atherosclerotic disease manifestations, e.g. acute myocardial infarction and stroke [5,6].

The major source of IL-1β and TNF-α in atherosclerotic lesions is macrophages, derived from blood monocytes infiltrating the subintima [7]. Secretion of biologically active IL-1β from monocytes and macrophages requires two activation signals. Signal 1 is delivered through toll-like receptors (TLRs) and leads to transcription of pro-IL-1β, pro-IL-18 [8] and pro-IL-33 [9]. Signal 2 is delivered by danger molecules, e.g. reactive oxygen species, and fungal, bacterial and viral pathogens, which bind to nucleotide-binding oligomerization domain-like receptors (NLRs) and cause assembly of multiprotein oligomers known as inflammasomes. Inflammasomes, in turn, activate caspase-1, which cleaves the precursor forms of the cytokines into active IL-1β and IL-18 [8,10]. CHCs have been shown to trigger inflammation by activation of the NLR family pyrin domain-containing 3 (NLRP3) inflammasome, leading to secretion of active IL-1β and TNF-α from monocytes and macrophages primed with TLR ligands, e.g. lipopolysaccharide (LPS) from *Escherichia coli*, cell wall components of Gram-positive bacteria, and oxidized low-density lipoprotein [11–13].

Much research has focused on pro-inflammatory conditions that facilitate the development of atherosclerotic disease. One such condition is PD, which affects up to 50% of the adult population over 50 years of age in industrialized countries [14,15]. Viable periodontal bacteria including *Porphyromonas gingivalis* have been found in atherosclerotic plaques in humans and in mice [16–18]. A considerable body of evidence has linked this bacterium to the pathogenesis of both PD and atherosclerotic CVD, but the underlying mechanisms remain unclear [19–21]. Oral infection with *P*. *gingivalis* and other bacteria induces secretion of IL-1β and TNF-α, as revealed in the crevicular fluid [22,23]. This promotes local tissue damage due to hyperinflammation, which may also cause systemic low-grade inflammation, and thereby increase the risk of atherosclerotic CVD [14]. Moreover, inflammation also causes ulceration in the periodontal pockets, thereby facilitating access of periodontal bacteria to the bloodstream, where they may spread suspended in plasma or attached to red blood cells (as is the case for *P*. *gingivalis*), thereby evading elimination by phagocytes [15,24], and subsequently gain access to the arterial wall and contribute to atherogenesis.

Recently, it has been shown that CHCs enhance the production of IL-1β by human macrophages primed with *P*. *gingivalis* LPS (Pg-LPS) [25]. However, results obtained with purified Pg-LPS may not apply to whole bacteria. Thus, gingipains, a family of cysteine proteases, constitute another major virulence factor of *P*. *gingivalis*, and are key players in the stimulation of bone matrix destruction and modulation of host responses in PD [26]. For example, the arginine (Arg) or lysine (Lys) specific gingipains of *P*. *gingivalis* may cleave CD14, a receptor for LPS [27], leading to LPS hyporesponsiveness [27]. While other studies have suggested that TLR2 is more important [28,29], recently Pg-LPS was shown to activate TLR4 which led to induction of pro-inflammatory cytokines in human gingival fibroblasts [30]. Regardless of the relative binding contribution of these TLRs, cleavage of CD14 by *P*. *gingivalis* gingipains may compromise signaling through both. Of note, gingipains *per se* stimulate the human monocytic cell line THP-1 for production of IL-8, IL-6, and monocyte chemotactic peptide (MCP)-1, in a process involving protease-activated receptors (PAR)-1, -2 and -3 [31], and human macrophages produce TNF-α after stimulation with both Arg- and Lys-gingipains [32].

In this study, we examined the ability of CHCs to stimulate unprimed and primed monocytes for production of the pro-inflammatory cytokines IL-1β, TNF-α and IL-6, the anti-inflammatory IL-10, and the chemokine IL-8. We also investigated the potential synergy between CHCs on the one hand, and Pg-LPS, Arg-gingipain and whole *P*. *gingivalis* bacteria on the other, in stimulation of these responses. In addition, we examined the role of inflammasomes in mediating *P*. *gingivalis*- and CHC-induced pro-inflammatory cytokine production.

## Materials and methods

### Cells

Blood from anonymous, healthy blood donors attending the Blood Bank at Copenhagen University Hospital, Rigshospitalet, Denmark was used. Peripheral blood mononuclear cells (PBMCs) were isolated using Lymphoprep (Axis-shield, Oslo, Norway) gradient centrifugation. PBMCs were resuspended in RPMI 1640 with HEPES (Biological Industries, Haemek, Israel), L-glutamine and gentamicin (Fischer Scientific, Slangerup, Denmark), and monocytes were isolated using EasySep^TM^ human CD14+ positive selection kit (STEMCELL technologies, Grenoble, France) according to the manufacturer’s instructions. Purity was shown to be >90% when tested by flow cytometry.

### Cholesterol crystal preparation

Cholesterol ≥99% (Sigma-Aldrich, St. Louis, MO, USA) was dissolved in 95% ethanol (12.5 g/L), heated to 60°C, filtered, and left at room temperature to allow crystallization in 15 mL polypropylene tubes (Nunc^TM^, Fishcer Scientific, Slangerup, Denmark). CHCs were collected by filtering and grinding using a sterile mortar. CHCs were stored in plastic tubes at -20°C until use. Any LPS contamination of CHCs was found to be below the detection limit of the *Limulus* amebocyte lysate assay QCL-1000 kit (Lonza, Walkersville, MD, USA).

### Stimulation of monocytes with LPS from *P*. *gingivalis* and *E*. *coli*, lipoteichoic acid, and Arg-gingipain

Freshly isolated CD14+ monocytes were cultured overnight in Nunclon Delta microwell plates (Thermo Fischer Scientific, Roskilde, Denmark) using 3x10^5^ cells/well with 200 μL RPMI 1640 with no serum. The cells were incubated with 10.0 μg/mL Ultrapure LPS from *P*. *gingivalis* (Pg-LPS) (Invivogen, San Diego, CA, USA), 0.01 and 1.0 μg/mL LPS from *E*. *coli* (Ec-LPS: O55:B5 endotoxin; Lonza, Walkersville, MN, USA), and 0.1 and 1.0 μg/mL the TLR2 agonist lipoteichoic acid from *Staphylococcus aureus* (Sa-LTA) (Invivogen, San Diego, CA, USA), in the presence or absence of 2 mg/mL CHCs. Furthermore, isolated monocytes were stimulated with Arg-gingipain (210 nM) (Hölzel Diagnostika Handels GmbH, Köln, Germany). Before use, Arg-gingipain was activated in RPMI 1640 and 10 mM cysteine for 10 minutes at 37°C/5% CO_2_, and then diluted in media. After 20 hours at 37°C/5% CO_2_, supernatants were harvested and analyzed for the presence of cytokines. No endotoxin was detected in the Arg-gingipain preparation.

### Cytokine measurements

IL-1β, IL-6, IL-10, and TNF-α were measured in supernatants from cell cultures using the BD Cytometric Bead Array Human Inflammation Kit (BD Bioscience, San Jose, CA, USA) as described previously [33]. Data acquisition was done with a FACSCalibur flow cytometer (BD Bioscience), and data were analyzed using the FCAPArray Software (Softflow, Burnsville, MN, USA). IL-8 in supernatants was measured using the Luminex100 detection system (Luminex Corporation, Austin, TX, USA) according to the manufacturer’s instructions.

### Blockade of TLR-2, TLR-4, the inflammasome, and IL-1 signaling

For blockade of TLR2 and TLR4, isolated monocytes were preincubated with 1 μg/mL monoclonal anti-TLR2 IgA (α-TLR2-IgA) (Invivogen, San Diego, CA, USA) or 2.5 μg/mL of the TLR4 antagonist LPS from *Rhodobacter sphaeroides* (Rs-LPS) (Invivogen, San Diego, CA, USA) for 30 minutes at 37°C/5% CO_2_ before stimulation with whole Pg bacteria. For blockade of inflammasome activity, 25 μM of the pan-caspase inhibitor Z-VAD-FMK (sc-3067, Santa Cruz Biotechnology, Santa Cruz), various concentrations of the caspase-1/NLRP3 inhibitor, Z-YVAD-FMK (Abcam, Cambridge, UK) (1, 10, and 100 μM), and the ATP-sensitive potassium channel inhibitor/NLRP3 inhibitor, glibenclamide (Invivogen, San Diego, CA, USA) (5, 25, and 50 μg/mL) were added. For blockade of IL-1 signaling, 2 ng/mL of the IL-1 receptor antagonist (IL-1RA) (Novus Biologicals, Cambridge, UK) was added.

### Stimulation of monocytes with whole bacteria

*P*. *gingivalis* (ATCC 33277) was cultured on trypticase soy blood agar plates containing 5 mg/mL hemin and 50 μg/L Vitamin K for 4 days at 37°C/10% CO_2_/10% H_2_O/80% N_2_. Subsequently, the bacteria were frozen in phosphate-buffered saline, pH 7.4, and kept at -80°C. Isolated CD14+ monocytes were stimulated overnight (3x10^5^ cells/well) with 4x10^5^ thawed whole bacteria in RPMI 1640 with no serum added. After 20 hours at 37°C/5% CO_2_, supernatants were harvested and analyzed for the presence of cytokines.

### Analyses of cell viability using flow cytometry

Purified CD14+ cells were stained with anti-CD14-antibody (BD Bioscience, San Jose, CA) and 7-Amino Actinomycin D (7-AAD) and incubated on ice for 30 minutes. Cells were analyzed using a FACS canto II flow cytometer (BD Bioscience, San Jose, CA) and data were analyzed using FlowJo v.X, (TreeStar, Inc. Ashland, OR).

### Statistical analyses

Statistical analyses were performed using GraphPad Prism 6.0 (GraphPad Software, CA, USA). Data are presented as means±SDs. Cytokine secretion upon stimulation with and without CHCs was compared using repeated measures ANOVA. Differences between paired data were assessed using paired sample t-tests. The Bonferroni correction was used where independent comparisons were made. Data were log10-transformed to obtain normal distribution when indicated. P<0.05 was considered significant.

## Results

### Monocyte cytokine responses to CHCs

**Fig 1:**
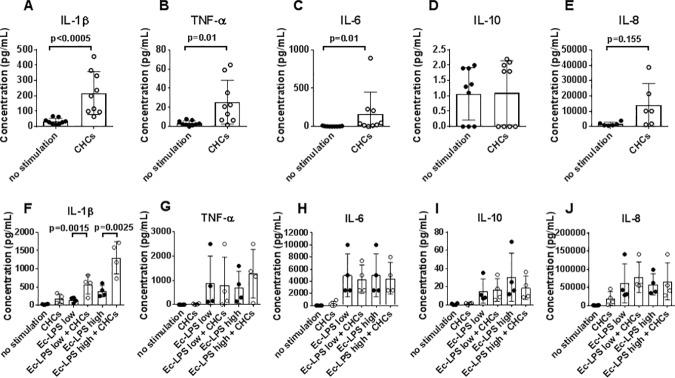
Influence of cholesterol crystals (CHCs) on *E*. *coli* (Ec)-lipopolysaccharide (LPS)-induced cytokine responses. (A–E) Isolated monocytes were stimulated with CHCs (2mg/mL) for 20 hours. Interleukin (IL)-1β, tumor necrosis factor (TNF)-α, IL-6, IL-10, and IL-8 in supernatants were measured after 20 hours of incubation, and are shown as mean±SD for experiments using 9 healthy donors. (F–J) Isolated monocytes were stimulated with Ec-LPS at two concentrations; 0.01 (low) and 1.0 (high) μg/mL in the absence (black circles) or presence (white circles) of CHCs (2mg/mL). Concentrations of IL-1β, TNF-α, IL-6, IL-10, and IL-8 in supernatants after 20 hours of incubation are shown as mean±SD for experiments using 4 healthy donors.

While examining the ability of CHCs to stimulate production of pro-inflammatory cytokines by unprimed human monocytes, we observed significant secretion of IL-1β, TNF-α, IL-6 (Fig 1A–1C), and a statistical trend towards IL-8 secretion (Fig 1E). The CHC concentration used (2 mg/mL) was based on preliminary titration studies (S1 Fig).

### Influence of CHCs on TLR4-mediated cytokine responses

We next examined the influence of CHCs on monocyte cytokine responses induced by LPS from *E*. *coli* (Ec-LPS), a classic TLR4 agonist (Fig 1). The CHCs enhanced IL-1β responses to Ec-LPS at two concentrations (0.01 and 1 μg/mL) (Fig 1F) without affecting the production of TNF-α, IL-6, IL-10, and IL-8 (Fig 1G–1J).

### Influence of CHCs on TLR2-mediated cytokine responses

**Fig 2:**
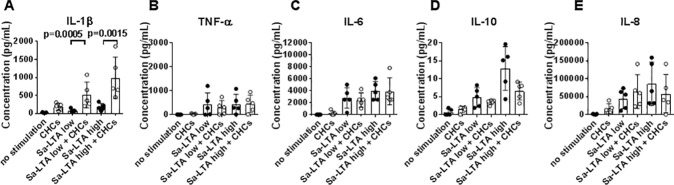
Influence of cholesterol crystals (CHCs) on toll-like receptor (TLR) 2-induced cytokine responses. (A–E) Isolated monocytes were stimulated with the TLR2 agonist lipoteichoic acid from *Staphylococcus aureus* (Sa-LTA) at two concentrations; 0.1 (low) and 1.0 μg/mL (high) in the absence (black circles) or presence (white circles) of CHCs (2 mg/mL). Concentrations of interleukin (IL)-1β, tumor necrosis factor (TNF)-α, IL-6, IL-10, and IL-8 in supernatants after 20 hours of incubation are shown as mean±SD for experiments using 5 healthy donors.

Further we examined the effect of CHCs on TLR2-induced cytokine responses by stimulating monocytes with the TLR2 agonist Sa-LTA. Dose-dependent secretion of IL-1β, IL-6, IL-10, and IL-8 was observed (Fig 2). Again, IL-1β secretion was enhanced by co-incubation with CHCs at 0.1 and 1.0 μg/mL of Sa-LTA (Fig 2A), while CHCs had no effect on secretion of the other cytokines (Fig 2B–2E).

### Influence of CHCs on cytokine responses to *P*. *gingivalis* LPS

Examination of the monocyte cytokine responses to LPS from *P*. *gingivalis* confirmed previous findings that TLR2 contributed to cytokine production induced by Pg-LPS [28,29] using a TLR2-blocking antibody (anti-TLR2, clone B4H2), which inhibited the production of IL-1β by 29±42%, TNF-α by 55±27%, IL-6 by 60±46%, and IL-10 by 54±45% (S2A–S2D Fig). Secretion of IL-8 was not affected by anti-TLR2 (S2E Fig).

**Fig 3:**
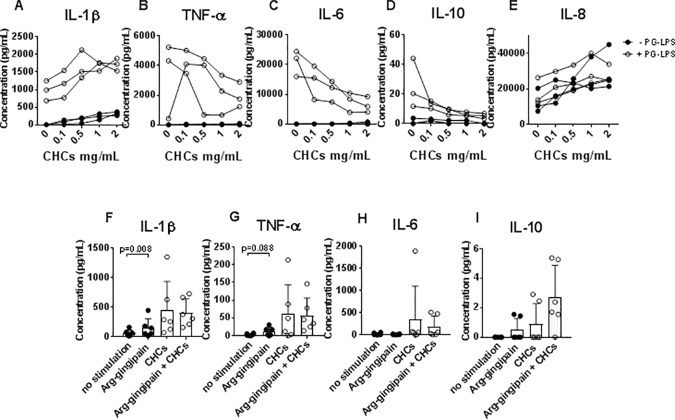
Influence of cholesterol crystals (CHCs) on cytokine responses induced by *P*. *gingivalis* lipopolysaccharide (Pg-LPS). (A–E) Isolated monocytes were stimulated with CHCs in different concentrations in the absence (black circles) or presence (white circles) of Pg-LPS (10 μg/mL). Concentrations of interleukin (IL)-1β, tumor necrosis factor (TNF)-α, IL-6, IL-10 and IL-8 in supernatants after 20 hours are shown as means±SD for experiments using 3 healthy donors. (F-I) Isolated monocytes were cultured with no stimulation, Arg-gingipain, CHCs, and Arg-gingipain and CHCs combined. The content of IL-1β, TNF-α, Il-6, and IL-10 in supernatants after 20 hours is shown as means±SD for experiments using 6 healthy donors.

Pg-LPS-induced secretion of IL-1β was greatly enhanced in a dose-dependent manner (Fig 3A) by co-stimulation with CHCs. In contrast, CHCs mediated a dose-dependent lowering of Pg-LPS-induced secretion of TNF-α, IL-6 and IL-10 (Fig 3B, 3C and 3D). Addition of CHCs did not affect the Pg-LPS induced secretion of IL-8 (Fig 3E).

### Influence of CHCs on cytokine responses to *P*. *gingivalis* gingipains

The cytokine production induced by another constituent of *P*. *gingivalis*, the cysteine protease Arg-gingipain activated by cysteine *in vitro* and added to monocyte cultures showed that Arg-gingipain induced secretion of IL-1β and TNF-α (Fig 3F and 3G), but not of IL-6, IL-10 (Fig 3H and 3I), and IL-8 (data not shown). Furthermore, CHCs did not influence IL-1β or TNF-α secretion (Fig 3F and 3G).

### Role of inflammasomes in cytokine responses to Pg-LPS and CHCs

To investigate the role of the inflammasome in the synergistic effect of Pg-LPS and CHCs on monocyte responses, the pan-caspase inhibitor Z-VAD-FMK was included in the experiments at a concentration of 25 μM, which was chosen on the basis of titration studies (S3A Fig). Z-VAD-FMK abolished secretion of IL-1β by monocytes stimulated with CHCs alone, confirming that CHCs induced IL-1β production by monocytes in an inflammasome-dependent manner (S3B Fig). Importantly, incubation of monocytes for 20 hours with Z-VAD-FMK at concentrations ranging from 5–25 μM did not cause significant cell death (S3C Fig). The enhancing effect of CHCs on Pg-LPS-induced IL-1β secretion by monocytes was abrogated by addition of Z-VAD-FMK (S3D Fig). In addition, production of TNF-α, IL-6, IL-10, and IL-8 after stimulation with Pg-LPS and CHCs was inhibited by Z-VAD-FMK (S3E–S3H Fig).

**Fig 4:**
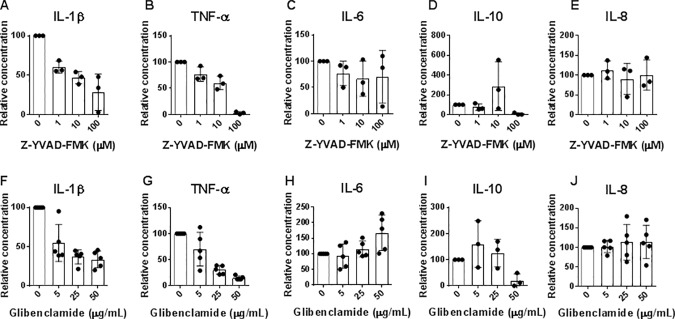
Role of the NLRP3 inflammasome in induction of cytokine responses to cholesterol crystals (CHCs) and *P*. *gingivalis* lipopolysaccharide (Pg-LPS). (A–E) Freshly isolated monocytes were cultured in presence of Pg-LPS (10 μg/mL) and CHCs, and the caspase-1 inhibitor Z-YVAD-FMK was added in concentrations of 5, 25 and 50 μg/mL. Concentrations of interleukin (IL)-1β, tumor necrosis factor (TNF)-α, IL-6, IL-10, and IL-8 in supernatants after 20 hours of incubation were measured, and data were normalized to *P*. *gingivalis*+CHCs. Data are shown as means±SD for experiments using 3 healthy donors. (F–J) Freshly isolated monocytes were cultured in presence of *P*. *gingivalis* lipopolysaccharide (Pg-LPS) (10 μg/mL) and CHCs (2 mg/mL), and the ATP-sensitive potassium channel inhibitor glibenclamide was added at concentrations of 1, 10 and 100 μM. Concentrations of IL-1β, TNF-α, IL-6, IL-10, and IL-8 in supernatants after 20 hours of incubation were measured, and data were normalized to *P*. *gingivalis*+CHCs. Data are shown as means±SD for experiments using 5 healthy donors.

To further investigate the role of the NLRP3 inflammasome, we included the caspase-1/NLRP3 inhibitor, Z-YVAD-FMK, and the ATP-sensitive potassium channel/NLRP3 inhibitor, glibenclamide in the experiments. Both Z-YVAD-FMK and glibenclamide caused a dose-dependent decrease in IL-1β secretion by monocytes stimulated with a combination of Pg-LPS and CHCs, confirming that NLRP3 was critically involved in the induction of IL-1β secretion (Fig 4A and 4F). In addition, secretion of TNF-α was lowered by both Z-YVAD-FMK and glibenclamide (Fig 4B and 4G), while the two NLRP3 inhibitors had no effect on the production of IL-6, or IL-8 (Fig 4C, 4D, 4E, 4H, 4I and 4J). The data on IL-10 were ambiguous (Fig 4D and 4I).

Addition of each inhibitor separately did not completely abolish secretion of IL-1β. However, addition of the inhibitors in combination at the highest concentration resulted in complete inhibition of IL-1β production (data not shown).

**Fig 5:**
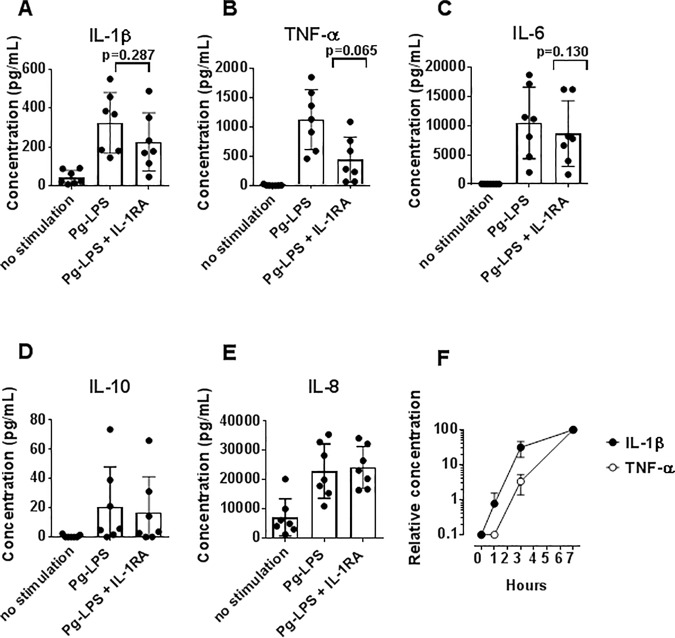
Effect of interleukin (IL)-1β inhibition on *P*. *gingivalis* lipopolysaccharide (Pg-LPS)-induced cytokine production. (A–E) Isolated monocytes were cultured without stimuli, or in the presence of Pg-LPS (10 μg/mL) or Pg-LPS in combination with IL-1 receptor antagonist (IL-1RA). Concentrations of interleukin (IL)-1β, tumor necrosis factor (TNF)-α, IL-6, IL-10, and IL-8 in supernatants after 20 hours are shown as means±SD for experiments using 7 healthy donors. (F) Isolated monocytes were cultured with Pg-LPS (10 μg/mL) and CHCs (2 mg/mL), and concentrations of IL-1β and TNF-α in supernatants were measured after 1, 3 and 7 hours of incubation. Data are shown as average and range for experiments using 2 healthy donors, normalized to the values obtained after 7 hours of incubation.

To examine the role of IL-1β for induction of TNF-α, IL-1 receptor antagonist (IL-1RA) was included in the experiments. IL-1RA did not affect secretion of IL-1β significantly (Fig 5A), but inhibited the production of TNF-α (Fig 5B), indicating a role for IL-1β in induction of TNF-α, as also observed by others [34]. In addition, IL-6 production was nonsignificantly lowered after addition of IL-1RA (Fig 5C). Secretion of IL-10 and IL-8 was not affected by addition of IL-1RA (Fig 5D and 5E).

The notion that secretion of TNF-α was a consequence of stimulation with IL-1β was supported by the finding that IL-1β secretion production preceded TNF-α secretion by approximately 3 hours (Fig 5F).

### Influence of CHCs on cytokine responses to whole bacteria

**Fig 6:**
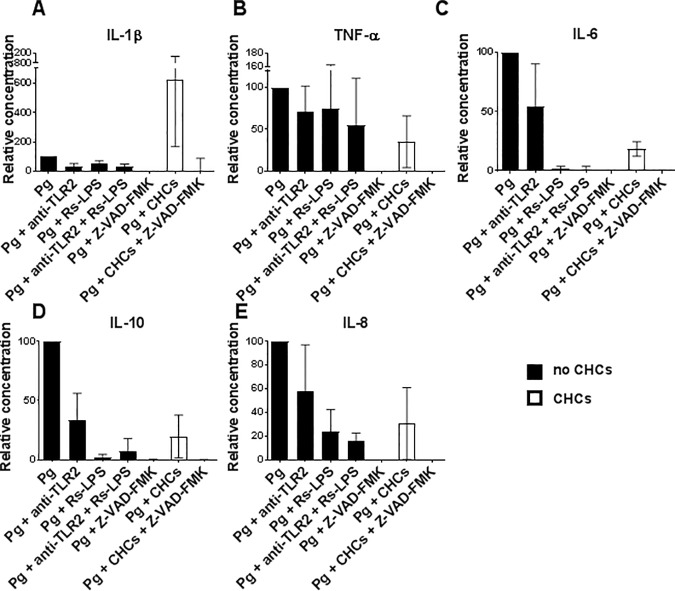
Toll-like receptor (TLR) 2-, TLR4- and caspase-dependent cytokine secretion of monocytes stimulated with *P*. *gingivalis* and cholesterol crystals (CHCs). (A–E) Isolated monocytes incubated with *P*. *gingivalis* (Pg), as whole bacteria, and CHCs in the presence of various combinations of an antibody blocking TLR2 (anti-TLR2), the TLR4 antagonist lipopolysaccharide (LPS) from *Rhodobacter sphaeroides* (Rs-LPS), and the pan-caspase inhibitor Z-VAD-FMK. Supernatant concentrations of interleukin (IL)-1β, tumor necrosis factor (TNF)-α, IL-6, IL-10, and IL-8 after 20 hours of incubation are shown as means±SD for experiments using 4 healthy donors. Background values obtained in the absence of stimuli were subtracted, and data normalized to values obtained after incubation with *P*. *gingivalis* alone.

Incubation of monocyte cultures with *P*. *gingivalis* as whole bacteria led to production of IL-1β, TNF-α, IL-6, IL-10, and IL-8 (Fig 6A–6E). Addition of anti-TLR2 and Rs-LPS, separately or in combination, lowered secretion of all cytokines (Fig 6A–6E), demonstrating the involvement of both TLR2 and TLR4 in their induction. Anti-TLR2 and Rs-LPS inhibited the production of IL-1β by 65±18% and 44±17%, respectively, with no additive effect of their combined use (data not shown).

The pan-caspase inhibitor Z-VAD-FMK completely abolished secretion of IL-1β, TNF-α, IL-6, IL-10, and IL-8 (Fig 6A–6E), demonstrating a critical role for inflammasomes in induction of all these cytokines by whole bacteria.

As observed for Pg-LPS (Fig 3A), CHCs markedly enhanced the secretion of IL-1β induced by whole bacteria (Fig 6A). In contrast, CHCs lowered the secretion of TNF-α, IL-6, IL-10, and IL-8 (Fig 6B–6E). Secretion of IL-1β, TNF-α, IL-6, IL-10, and IL-8 induced by *P*. *gingivalis* and CHCs was abrogated by Z-VAD-FMK, demonstrating the critical role of inflammasomes, also under these conditions (Fig 6A–6E).

## Discussion

Accumulation of CHCs plays a crucial role in atherogenesis, possibly via induction of pro-inflammatory cytokine responses through inflammasomes [5,6,11,12,25]. Infection with *P*. *gingivalis* which is thought to be a keystone pathogen in PD [20], has also been linked to the pathogenesis of atherosclerotic CVD [14,15,19–21]. We therefore assessed the ability of CHCs to stimulate human monocytes for secretion of pro-inflammatory cytokines and to enhance pro-inflammatory cytokine responses to *P*. *gingivalis*, and we examined the role of inflammasomes and TLRs in these processes.

CHCs alone were capable of inducing monocyte secretion of IL-1β, an important inflammatory mediator in CVD and PD [3,35,36]. Similar observations were made by Rajamäki et al., who used human adherent mononuclear cells and monocyte-derived macrophages [37]. Two other studies, however, found that CHCs alone did not induce IL-1β secretion by human PBMCs [12] or monocyte-derived macrophages [25]. These discrepancies may be explained by differences in incubation time (20 hours in our study versus 6 hours [12] and 6 days [25]), and the fact that we did not include GM-CSF or M-CSF [25]. We further observed that CHCs (2 mg/mL) alone induced secretion of TNF-α, IL-6, and IL-8 by monocytes. We recently found that matrix-bound cholesterol failed to stimulate monocyte-derived spindle-shaped cells for cytokine production [13], and this discrepancy may be due to differences in cholesterol formulation and differentiation stage of the cell cultures.

We show here that CHCs markedly enhance secretion of IL-1β by monocytes stimulated with the TLR4-ligand LPS from *E*. *coli*, supporting similar findings by Rajamäki et al. [37]. The Ec-LPS-induced production of TNF-α was not affected, however. The reverse pattern was found in our previous study using matrix-bound cholesterol [13]. CHCs also enhanced the secretion of IL-1β by monocytes stimulated with the TLR2-ligand lipoteichoic acid from *S*. *aureus*.

A main focus of this study was to examine whether *P*. *gingivalis* and CHCs acted synergistically in stimulation of pro-inflammatory cytokine production by monocytes, a molecular mechanism that could contribute to the association between PD and atherosclerosis. Indeed, CHCs markedly enhanced secretion of IL-1β induced by LPS isolated from *P*. *gingivalis*, in accordance with results of a recent study of human macrophages [25]. However, CHCs did not enhance the Pg-LPS-induced production of TNF-α or IL-6. Another component of *P*. *gingivalis*, Arg-gingipain, which is considered a key virulence factor, induced secretion of IL-1β and TNF-α, but not IL-6, IL-8 and IL-10. Similarly, previous studies showed that gingipains induced IL-8 and TNF-α production by human macrophages [32] and IL-1β and IL-10 production by the human monocytic cell line THP-1 [38]. Notably, CHCs did not enhance Arg-gingipain-mediated secretion of IL-1β by monocytes.

To our knowledge, this is the first study to assess the combined effect of CHCs and whole *P*. *gingivalis* bacteria on cytokine production by monocytes. *P*. *gingivalis* alone was a potent inducer of IL-1β, TNF-α-, IL-6-, and IL-10 secretion, as also described previously [39,40], and of IL-8 secretion, respectively, and TLR2 and TLR4 both contributed to *P*. *gingivalis-*mediated induction of cytokine responses. Notably, CHCs enhanced *P*. *gingivalis-*induced secretion of IL-1β by monocytes, but lowered the concomitant secretion of TNF-α, IL-6, IL-8, and IL-10. The underlying mechanisms and the consequences of these responses with respect to atherosclerotic plaque development and stability remain to be studied.

The pan-caspase inhibitor Z-VAD-FMK abolished the ability of CHCs to induce IL-1β secretion by monocytes and neutralized the CHC-mediated enhancement of IL-1β production induced by Pg-LPS and whole *P*. *gingivalis*. This implied that CHCs exerted their effects on IL-1β production via caspase activation, and thereby through inflammasomes. It has previously been demonstrated that CHCs activate the NLRP3 inflammasome in Ec-LPS stimulated macrophages [37] and human PBMC [12]. Here, we investigated the role of the NLRP3 inflammasome in the enhancement of IL-1β secretion induced by Pg-LPS and CHCs by use of the NLRP3 inhibitors, Z-YVAD-FMK and glibenclamide. We demonstrated that the NLRP3 inflammasome is involved in the CHC-mediated enhancement of IL-1β production by monocytes stimulated with LPS from the periodontal bacterium *P*. *gingivalis*.

In line with the findings of others [34], our experiments including IL-1RA showed that IL-1β enhanced the secretion of TNF-α, IL-6 and IL-10, but not IL-8. Also, IL-1β secretion began approximately 3 hours before TNF-α secretion.

Among the limitations of the study, it should be noted that we isolated monocytes based on their expression of CD14, which is also expressed by macrophages. The cell cultures may therefore have contained macrophages at low frequencies.

In summary, we demonstrated an inflammasome-dependent role for CHCs in enhancement of the pro-inflammatory cytokine responses of human monocytes exposed to the pro-atherogenic periodontal bacterium *P*. *gingivalis*. This interaction may contribute to the association between PD and atherosclerotic CVD.

## Supporting information

S1 Fig Dose-titration of cholesterol crystals (CHCs). Isolated monocytes were cultured with no stimulation and CHCs at different concentrations (0.1, 0.5, 1, 2 and 4 mg/mL as indicated by numbers after CHCs). The content of interleukin (IL)-1β in the supernatants after 20 hours is shown as means±SD for experiments using two healthy donors.
(TIF)

S2 Fig The role of toll-like receptors (TLR) 4 and TLR2 in cytokine responses upon stimulation with *E. coli* (Ec)-lipopolysaccharide (LPS) and *P. gingivalis* (Pg)-LPS. (A-E) Isolated monocytes were cultured with no stimulation, anti-TLR2, Pg-LPS) alone and Pg-LPS in combination with anti-TLR2 antibody. The content of IL-1β (n = 11), TNF-α (n = 11), IL-6 (n = 11), IL-10 (n = 10) and IL-8 (n = 7) in the supernatants after 20 hours is shown as means±SD. P-values were calculated from log10-transformed data using paired t-test.
(TIF)

S3 Fig Dose titration of the pan-caspase inhibitor Z-VAD-FMK. (A) Isolated monocytes were cultured with no stimulation, *P*. *gingivalis* (P.g)-lipopolysaccharide (LPS) and cholesterol crystals (CHCs), and Pg-LPS and CHCs in combination with different concentrations of Z-VAD-FMK (5, 10 and 25μM). Concentrations of interleukin (IL)-1β in the supernatants after 20 hours are shown as means±SD for results of experiments using two healthy donors. (B) Isolated monocytes were cultured with no stimulation, CHCs (2mg/mL) alone and in combination with Z-VAD-FMK (25μM). Concentrations of IL-1β in the supernatants after 20 hours are shown as means±SD for experiments using 10 healthy donors. (C) Isolated monocytes were cultured in presence of different concentrations of Z-VAD-FMK (5, 10, 25μM), Pg-LPS (10 μg/mL), and Pg-LPS in combination with Z-VAD-FMK (25 μM). After 20 hours, cells were stained with the dead cell marker 7-AAD and analyzed by flow cytometry. Frequencies of living CD14+ cells are shown for experiments using two healthy donors. (D-H) Freshly isolated monocytes were cultured in presence of Pg-LPS (10 μg/mL), Pg-LPS in combination with CHCs, or Pg-LPS in combination with both CHCs and the pan-caspase inflammasome-inhibitor Z-VAD-FMK. Concentrations of IL-1β, tumor necrosis factor (TNF)-α, IL-6, IL-10, and IL-8 in supernatants after 20 hours are shown as mean±SD for experiments using seven healthy donors.
(TIF)
