# Supplementary figures and images for: Cholesterol crystals enhance TLR2- and TLR4-mediated pro-inflammatory cytokine responses of monocytes to the proatherogenic oral bacterium Porphyromonas gingivalis
Source: PLoS One. 2017 Feb 24;12(2):e0172773. doi: 10.1371/journal.pone.0172773 (PMC5325525; doi:10.1371/journal.pone.0172773)

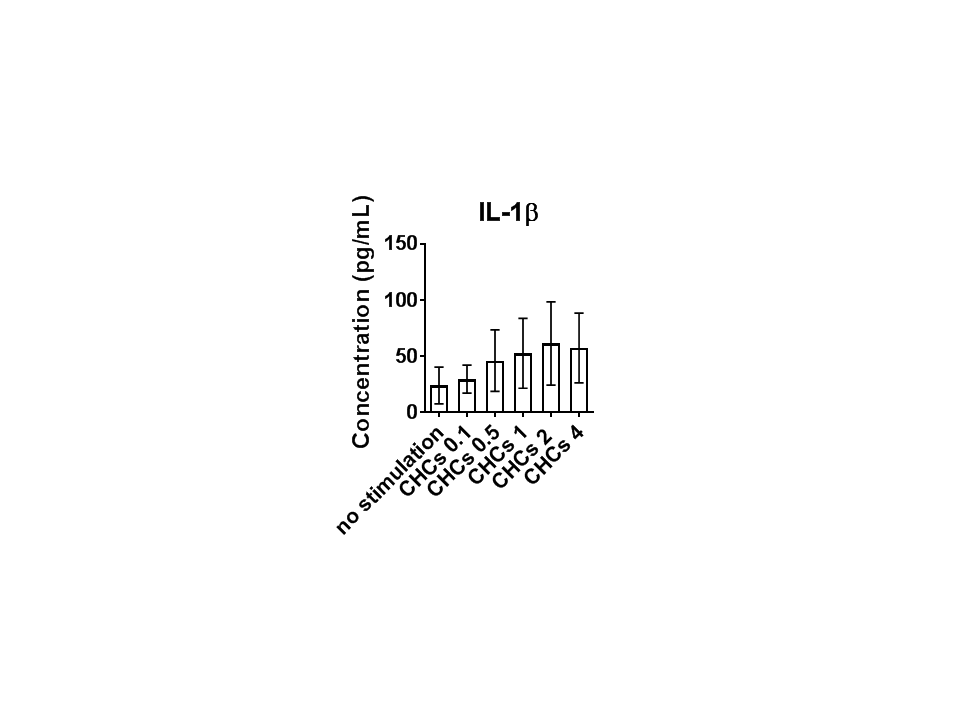

Supplement: S1 Fig — Isolated monocytes were cultured with no stimulation and CHCs at different concentrations (0.1, 0.5, 1, 2 and 4 mg/mL as indicated by numbers after CHCs). The content of interleukin (IL)-1β in the supernatants after 20 hours is shown as means±SD for experiments using two healthy donors. (TIF) [file pone.0172773.s001.tif]

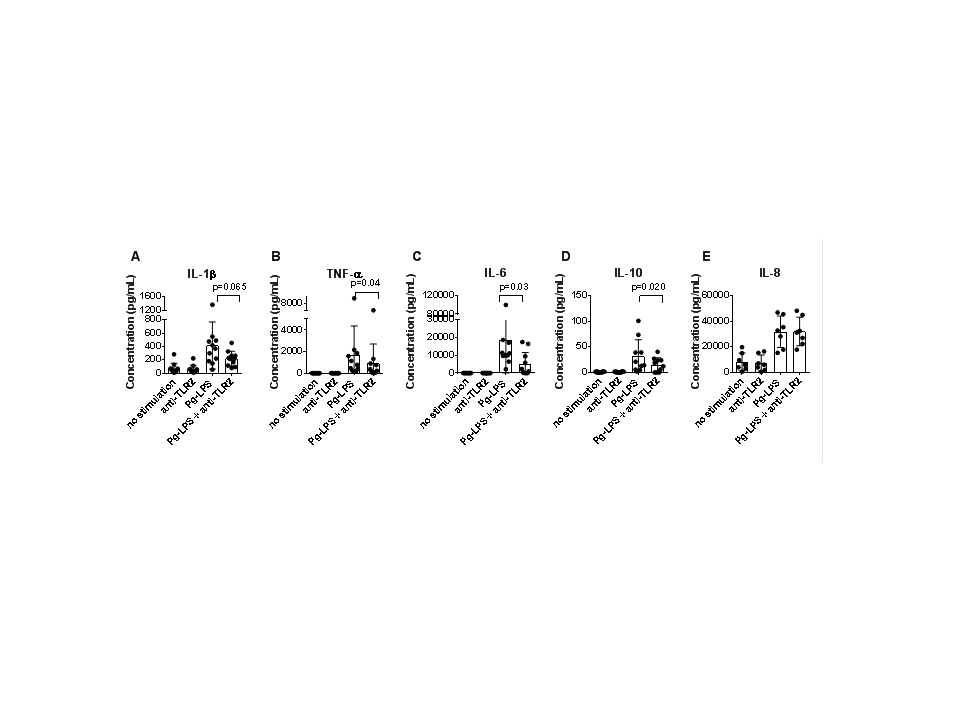

Supplement: S2 Fig — (A-E) Isolated monocytes were cultured with no stimulation, anti-TLR2, Pg-LPS) alone and Pg-LPS in combination with anti-TLR2 antibody. The content of IL-1β (n = 11), TNF-α (n = 11), IL-6 (n = 11), IL-10 (n = 10) and IL-8 (n = 7) in the supernatants after 20 hours is shown as means±SD. P-values were calculated from log10-transformed data using paired t-test. (TIF) [file pone.0172773.s002.tif]

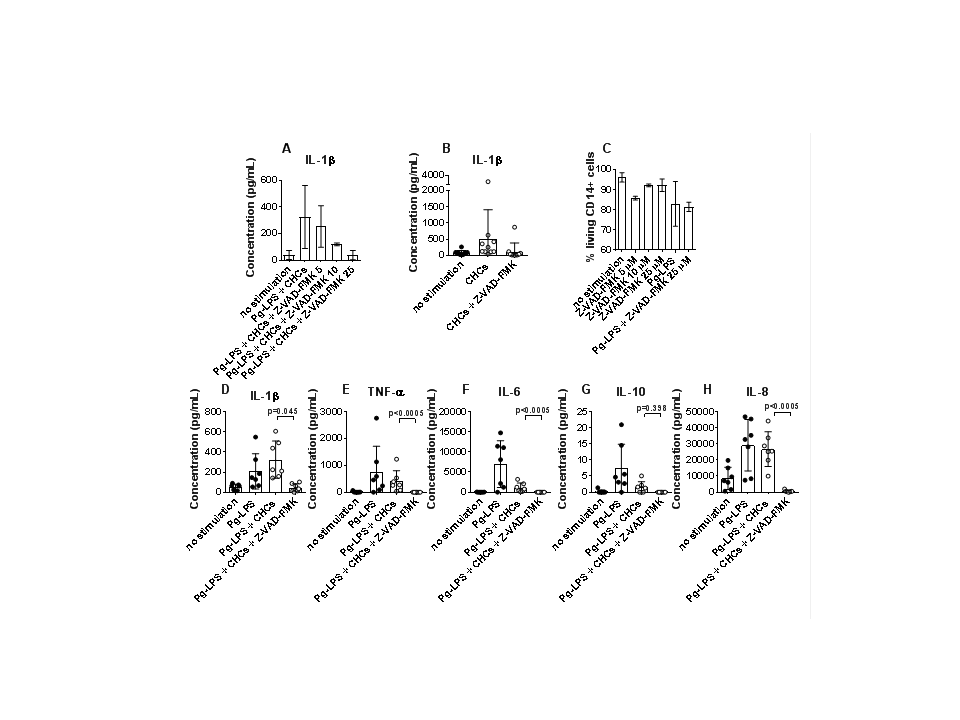

Supplement: S3 Fig — (A) Isolated monocytes were cultured with no stimulation, P. gingivalis (P.g)-lipopolysaccharide (LPS) and cholesterol crystals (CHCs), and Pg-LPS and CHCs in combination with different concentrations of Z-VAD-FMK (5, 10 and 25μM). Concentrations of interleukin (IL)-1β in the supernatants after 20 hours are shown as means±SD for results of experiments using two healthy donors. (B) Isolated monocytes were cultured with no stimulation, CHCs (2mg/mL) alone and in combination with Z-VAD-FMK (25μM). Concentrations of IL-1β in the supernatants after 20 hours are shown as means±SD for experiments using 10 healthy donors. (C) Isolated monocytes were cultured in presence of different concentrations of Z-VAD-FMK (5, 10, 25μM), Pg-LPS (10 μg/mL), and Pg-LPS in combination with Z-VAD-FMK (25 μM). After 20 hours, cells were stained with the dead cell marker 7-AAD and analyzed by flow cytometry. Frequencies of living CD14+ cells are shown for experiments using two healthy donors. (D-H) Freshly isolated monocytes were cultured in presence of Pg-LPS (10 μg/mL), Pg-LPS in combination with CHCs, or Pg-LPS in combination with both CHCs and the pan-caspase inflammasome-inhibitor Z-VAD-FMK. Concentrations of IL-1β, tumor necrosis factor (TNF)-α, IL-6, IL-10, and IL-8 in supernatants after 20 hours are shown as mean±SD for experiments using seven healthy donors. (TIF) [file pone.0172773.s003.tif]
